# Supplementary material for: Detergent-Triggered Membrane Remodelling Monitored via Intramembrane Fluorescence Dequenching
Source: ACS Omega. 2026 Jan 8;11(3):4465–74. doi: 10.1021/acsomega.5c10435 (PMC12854627; doi:10.1021/acsomega.5c10435)
Supplement: Supplementary file 1 [file ao5c10435_si_001.pdf]

**Detergent-Triggered Membrane Remodelling Monitored via Intramembrane Fluorescence Dequenching**

Claudia M. F. Andrews[1], Christopher M. Hofmair[1], Lauryn Roberts[1], Emily James[1], Katie Morris, Kevin Kramm[2], Mark C. Leake[1,3,4], Yue Wang[1], & Steven D. Quinn[1,4*]

[1] School of Physics, Engineering and Technology, University of York, Heslington, York, UK. YO10 5DD.

[2]PicoQuant, Rudower Chaussee 29, 12849, Berlin, Germany.

[3]Department of Biology, University of York, Heslington, York, UK. YO10 5DD.

[4]York Biomedical Research Institute, University of York, Heslington, York, UK. YO10 5DD

*steven.quinn@york.ac.uk

# Supporting Information

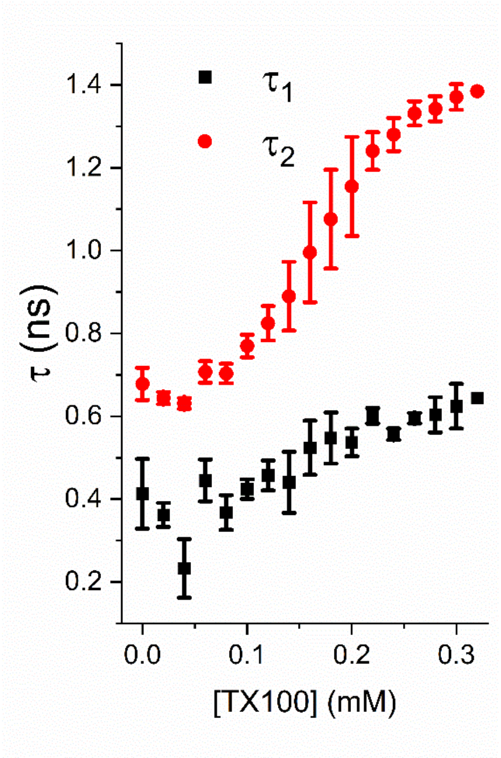


**Figure S1**. Representative variation in fast ($\tau_1$)and slow ($\tau_2$) lifetime components obtained from POPC vesicles containing 1 % DiI across the titration. Data points represent the mean and standard error of the mean from three separated experimental runs. Solution conditions: 50 mM Tris, pH 8.

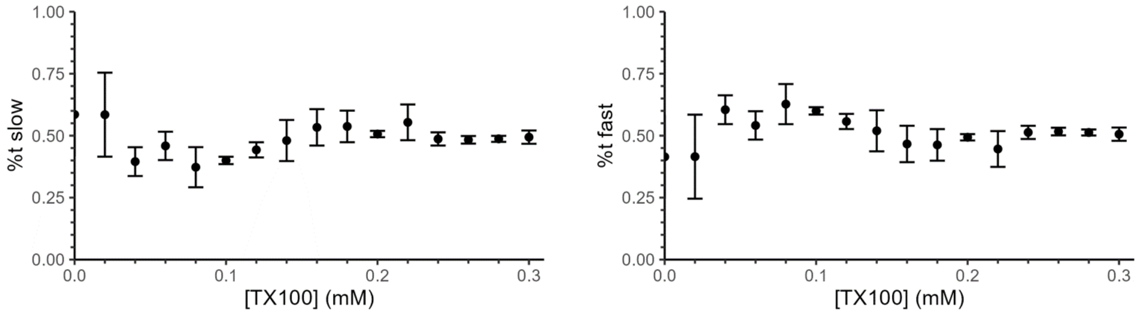


**Figure S2**. Representative variation in % contributions of the fast and slow lifetime components obtained from POPC vesicles containing 1 % DiI across the titration. Data points represent the mean and standard error of the mean from three separated experimental runs. Solution conditions: 50 mM Tris, pH 8.

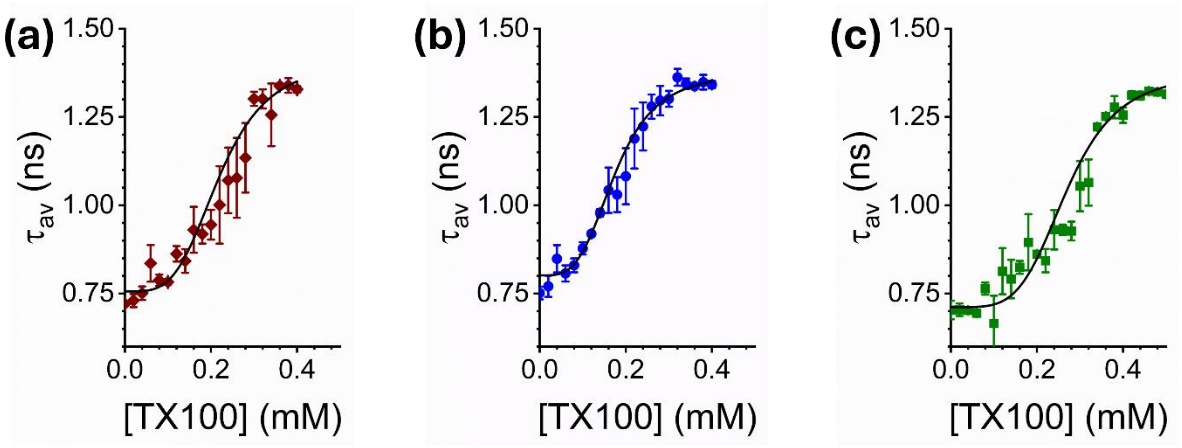


**Figure S3. Fluorescence dequenching applied to vesicles of various size.** Variation in $\tau_{av}$ obtained from (a) 30 nm (red, k = 0.22 ± 0.02 mM, n = 3.8 ± 0.9, $\chi^2$ = 0.98), (b) 400 nm (blue, k = 0.18 ± 0.01 mM, n = 3.6 ± 0.4, $\chi^2$ = 0.99) and (c) 1000 nm (green, k = 0.25 ± 0.01 mM, n = 4.5 ± 0.5, $\chi^2$ = 0.98) sized vesicles labelled with 1 % DiI in the absence and presence of TX-100. Solid black lines represent the corresponding Hill fits. In all cases, data points represent the mean values from three separate experimental runs and error bars denote the standard error of the mean. Solution conditions: 50 mM Tris, pH 8.

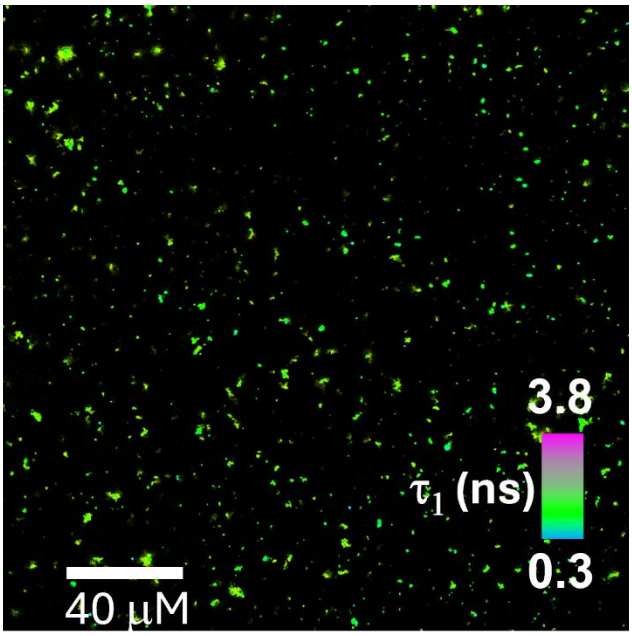


**Figure S4.** Representative FLIM image of surface-immobilized POPC vesicles incorporating 1 % DiI after addition of 0.15 mM TX-100 showing variations in fast component, $\tau_1$, across the field of view.

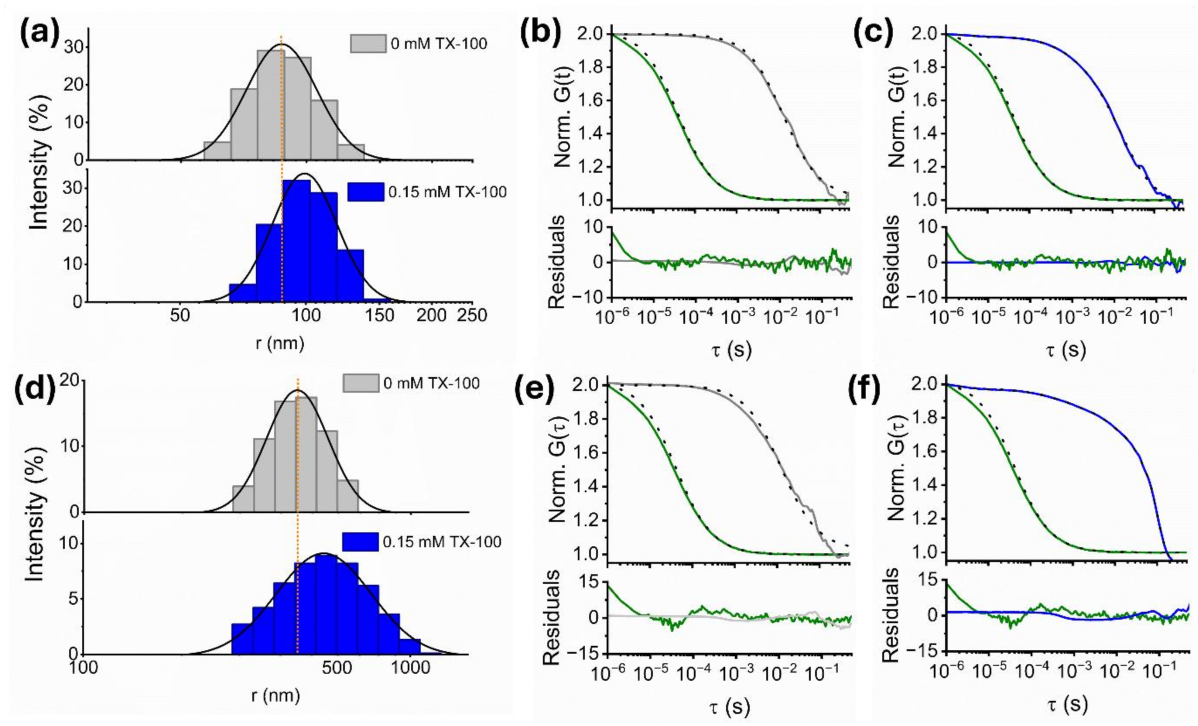


**Figure S5. Variations in vesicle size reported by DLS and FCS.** (a) Representative hydrodynamic radius (r) distributions obtained from POPC vesicles (1 % DiI) extruded through 200 nm pores in the absence (top panel) and presence (bottom panel) of 0.15 mM TX-100. The solid black lines represent lognormal fits to the experimental data. Also shown are normalized FCS correlation curves obtained from the ~200 nm sized vesicles in the (b) absence (grey, top panel) and (c) presence (blue, top panel) of TX-100. Solid green lines represent the normalized FCS curves obtained from Cy3B, and dashed black lines represent corresponding fits to the experimental data. Bottom panels: residuals of the fits. (d) DLS radii distributions obtained from POPC vesicles (1 % DiI) extruded through 1 μm pores in the absence (top panel) and presence (bottom panel) of 0.15 mM TX-100. The solid black lines represent lognormal fits to the experimental data. Also shown are normalized FCS correlation curves obtained from the ~1000 nm sized vesicles in the (e) absence (grey, top panel) and (f) presence (blue, top panel) of TX-100. Solid green lines represent the normalized FCS curves obtained from Cy3B, and dashed black lines represent corresponding fits to the experimental data. Bottom panels: residuals of the fits.

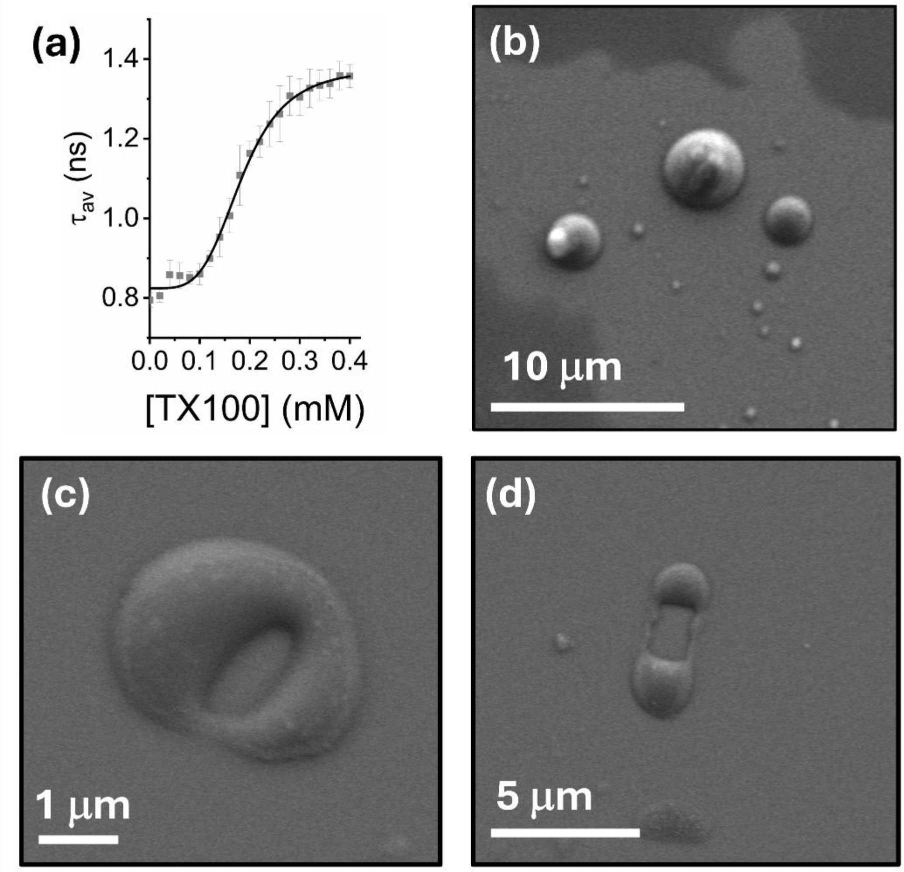


**Figure S6. TX-100 induces vesicle swelling and morphological alterations in giant unilamellar vesicles.** (a) Representative variation in $\tau_{av}$ as TX-100 was progressively added to freshly prepared giant unilamellar vesicles (GUVs) containing 1 % DiI. The solid black line represents a Hill fit to the experimental data ($\chi^2$ = 0.99; k = 0.18 ± 0.01 mM). (b) Representative SEM images of freshly prepared GUVs in the absence and (c,d) presence of 0.3 mM TX-100.
